# Content analysis of on-package formula labelling in Great Britain: use of marketing messages on infant, follow-on, growing-up and specialist formula

**DOI:** 10.1017/S1368980023000216

**Published:** 2023-08

**Authors:** Rana Conway, Sara Esser, Andrew Steptoe, Andrea D Smith, Clare Llewellyn

**Affiliations:** 1 Research Department of Behavioural Science and Health, University College London, Gower Street, London, WC1E 6BT, UK; 2 MRC Epidemiology Unit, University of Cambridge, Cambridge, UK

**Keywords:** Infant formula, BMS, Marketing, Policy, Child nutrition, Labelling

## Abstract

**Objective:**

To explore on-package formula messaging with reference to legislation and government-issued guidance in Great Britain (GB).

**Design:**

Formula products were identified, pictures of all sides of packs collated and on-package text and images were coded. Compliance with both GB legislation and guidance issued by the Department of Health and Social Care (DHSC) was assessed.

**Setting:**

All formula packs that were available for sale over the counter in GB between April and October 2020.

**Participants:**

Formula packs (*n* 71) including infant formula, follow-on formula, growing-up formula and specialist formula were identified, coded and analysed.

**Results:**

In total, 41 % of formula packs included nutrition claims, and 18 % included health claims that may be considered non-permitted, according to DHSC guidance. Additionally, 72 % of products showed images considered ‘non-permitted’. Breast Milk Substitute (BMS) legislation states infant and follow-on formula packs should be clearly distinguishable but does not provide criteria to assess similarity. Based on DHSC guidance, 72 % of infant and follow-on formula packs were categorised as showing a high degree of similarity. Marketing practices not covered by current legislation were widespread, such as 94 % of infant formula packs including advertisements for follow-on or growing-up formula.

**Conclusions:**

Text and images considered non-permitted according to DHSC guidance for implementing BMS legislation were widespread on formula products available in GB. As terms such as ‘similarity’ are not defined in BMS legislation, it was unclear if breaches had occurred. Findings support the WHO call for loopholes in domestic legislation to be closed as a matter of urgency.

Breastfeeding makes an important contribution to infant and maternal health^(1),^ and exclusive breastfeeding is recommended by the WHO for the first 6 months, with the introduction of complementary foods and continued breastfeeding thereafter^(2)^. Breastfeeding rates vary considerably across the globe, with just 1% of infants being exclusively breastfed for 6 months in the UK, compared to much higher rates in other high-income countries such as the USA (19 %) and Netherlands (18 %)^(3)^. Marketing and promotion of Breast Milk Substitutes (BMS) have an important influence on feeding decisions^(4)^ as products are presented as the ‘normal’ or ‘ideal’ food for infants, rather than as a specialist product to be given if breastfeeding is not possible. To protect public health, the promotion of BMS is restricted by EU regulation No 609/2013^(5)^ and delegated acts^(6,7)^, which give effect to the principles and aims of the WHO International code of Marketing of Breast-Milk Substitutes (‘the Code’) and its subsequent resolutions^(8)^. These regulations are intended to protect the public from inappropriate and potentially harmful promotion of BMS^(8)^ so as not to discourage breastfeeding^(5)^. The regulations cover infant formula (IF), follow-on formula (FOF) and specialist formula labelled as a Food for Special Medical Purposes (FSMP) but exclude growing-up formula (GUF).

UK guidance aligns with WHO recommendations regarding exclusive breastfeeding for the first 6 months and promotes continued breastfeeding for at least the first year of life^(9)^. If infants are not breastfed, it is recommended they are fed IF for the first year of life and then move on to full-fat cows’ milk^(9,10)^. Following EU exit, all three countries in GB adopted EU legislation covering BMS marketing and promotion^(11–13)^. To guide companies manufacturing or selling IF and FOF to implement the legislation, the Department of Health and Social Care (DHSC) issued guidance in 2013^(14)^ and 2021^(15)^ (Table 1). The guidance sets out DHSC’s interpretation of the requirements of the legislation as they apply in England, while recognising the principles are similar throughout GB^(14,15)^. While the guidance is intended to ‘facilitate adherence to and assessment of adherence to the legislation’, the DHSC highlight it ‘should not be taken as an authoritative statement on how the law should be interpreted’^(14,15)^. The legislation sets out in broad terms the text and imagery that is prohibited from labels, such as forbidding the use of ‘pictures or text which may idealise the use’ of formula^(5)^. DHSC guidance by contrast provides greater detail, such as examples of pictures that should be avoided, e.g. ‘toys, cots or young animals’^(15)^. Table 1 provides further examples of the scope of the legislation and corresponding guidance from DHSC.

**Table 1:** Key features of formula legislation relevant to mapping exercise and summary of guidance setting out DHSC’s interpretation of the requirements of the legislation

Topic	Legislation^(5,6,7)^	DHSC guidance for 2007 legislation^(14)^	DHSC guidance for 2013 legislation^(15)^*	Summary findings
Nutrition & Health Claims	Nutrition & Health Claims are prohibited on IF† Claims can be made for nutrients present in sufficient quantities if likely to be understood by consumers^(31)^. Only claims included in the GB NHC Register are permitted.	Only claims meeting criteria in 2007 legislation are permitted on IF.	Claims that should be considered ‘non-permitted’ on IF include ‘contains all the nutrients your baby needs to grow strong and healthy’, ‘easy to digest’ and ‘gentle’.	44 % of FOF included nutrition claims considered non-permitted. 17 % of IF and 17 % of FOF included health claims considered ‘non-permitted’.
DHA claim	A statement regarding DHA is permitted on IF, providing it is made clear DHA is present in all infant formula on the market e.g. ‘contains DHA (as required by the legislation for all infant formula)’.	Not covered in 2007 legislation therefore not included in guidelines. Food labelling legislation notes it is likely to confuse consumers if some nutrition information is partly on the front of pack and partly on the back^(47)^.	The DHA statement should be in close proximity to the area of the packaging, highlighting the presence of DHA.	0 % of IF making a DHA claim gave equal prominence to the statement about DHA addition being mandatory.
Similarity between IF and FOF	Packaging (text, images and colours) should be designed to avoid risk of confusion.	Using the same images and blocks of text in the same position is likely to confuse consumers. Colour schemes should be different, not just different shades of the same colour.	IF and FOF must differ in relation to text, images and colours used on packaging. DHSC does not consider different shades of the same colour to be an appropriate difference.	72 % of FOF were almost identical to same brand IF (similarity scored ≥4 out of 5).
Similarity between FSMP and IF	As above	Not covered in 2007 legislation therefore not mentioned in this guidance.	Not covered in Commission Delegated Regulation (EU) 2016/127 and therefore not in this guidance.	29 % of FSMP formula and 56 % of other specialist formulas were almost identical to IF (similarity score ≥4 out of 5).
Important Notice	A statement concerning the superiority of breastmilk and recommending use only on advice of an independent expert should be included. This should be preceded by the words ‘important notice’.	The important notice should be afforded a high degree of prominence. It should be clearly visible and understandable.	The important notice should be afforded a high degree of prominence. It should be clearly visible and understandable.	100 % of formulas included the Important Notice in small text that was difficult to locate.
Text discouraging breastfeeding	Label should not use terms that might idealise the use of formula e.g. humanised, maternalised, adapted or similar.	Non-mandatory text should not refer to breastmilk, breastfeeding, moving on from breastfeeding, closer to/inspired by breastmilk. Terms such as ‘the best’ or ’ideal method’ of infant feeding should not be used.	Non-mandatory text should not refer to breastmilk, breastfeeding, moving on from breastfeeding, closer to/inspired by breastmilk.	6 % of IF and 22 % FOF included text that could be considered ‘non-permitted’
Images discouraging breastfeeding	Label should not include pictures of infants or other images that could idealise the use of formula.	Images that could idealise the use of formula include toys, cots, young animals and graphics representing nursing mothers.	Images that could idealise the use of formula include graphics that represent nursing mothers, baby or child-related subjects and anthropomorphic characters, pictures and logos.	67 % of IF and 78 % FOF showed images that could be considered ‘non-permitted’

DHSC, Department of Health and Social Care; IF, infant formula (from birth); FOF, follow-on formula (6+months); GUF, growing-up formula (approximately 12+ months); FSMP, food for special medical purposes (from birth).

* Guidance for most recent legislation (Commission Delegated Regulation (EU) 2016/127) was not available at the time of analysis.

† With the exception of DHA, providing it is made clear DHA addition is mandatory.

There is more leeway for labelling of FOF and GUF than for labelling IF insofar as they are allowed to include nutrition and health claims. As nutrition and health claims are recognised in the legislation as promotional tools, they are not permitted on IF^(3,6)^. Likewise, FOF and GUF can be marketed directly to parents, whereas direct-to-consumer promotion is not allowed for IF. One requirement with regards to the advertisement of FOF is that it must not cross-promote IF and must explicitly make it obvious that the product is for older infants. In line with this, the legislation states that labelling of FOF should be designed to make it easily distinguishable from IF.

The ingredient and nutrient content of IF and FOF are also strictly controlled by the legislation to ensure compositional changes supported by good evidence are applied to all products and, as a result, there are no significant nutrient differences between brands^(5,16)^. There is concern that on-package and other promotional messaging may mislead caregivers about the similarity of formula to breast milk and the superiority of one product over another. Research shows most caregivers trust marketing claims and that these beliefs shape infant feeding decisions, increase formula use and thereby may undermine advice or decisions to breastfeed^(17)^. Sophisticated marketing strategies are used to promote BMS and companies increasingly rely on implied claims, such as presenting breast milk and infant formula in the same sentence, or displaying images of natural rural scenes, as a mechanism to circumvent legislation and influence purchasing behaviours^(17,18)^.

Inappropriate BMS marketing online and via TV and magazines has been highlighted in a number of studies^(19–21)^. While these media are transient and can be difficult to monitor, on-package labelling is required to include certain mandatory information, and compliance with legislation is easier to enforce. The aim of the current study was to explore on-package formula messaging in GB, including the use of health and nutrition claims, and identify commonalities. Secondly, to compare messaging to formula labelling legislation and DHSC guidance for complying with the legislation.

## Methods

This study applied a two-step process to: (i) identify all IF, FOF, GUF and specialist formula available in GB between April and October 2020; and (ii) categorise on-package text and images according to the type of claim or message presented. Where applicable, compliance with GB formula regulations was assessed.

### Step one: identification of products

Formula products were identified using several approaches to maximise coverage. First, websites belonging to the ten supermarkets and four pharmacy chains with the largest market share were hand searched^(22,23)^. To ensure no brands or product lines had been omitted, researchers also viewed websites for formula brands, Kantar household purchasing data, First Steps Nutrition Trust reports^(24)^ and twenty shops were visited in person (London, UK) – see supplementary material. The search procedure was repeated throughout the period of data collection to identify any new brands or product lines.

The following inclusion criteria were applied when selecting formula products: labelled as appropriate from birth to age 3 years; powdered; cow, goat, sheep or soya based; available over the counter. All other formula products were excluded; for example, homemade formula, powdered milk not specifically labelled as appropriate for children under 3 years, formula for preterm or low birth weight infants, products only available by prescription and formula only available online. Initial product screening of ready-to-feed formula products found labels included no additional claims compared to the equivalent powder product, therefore these were also excluded.

When products were available in more than one pack size (e.g. 200 g and 800 g), then the Kantar dataset was used to select the most frequently purchased size, so that only one pack of a specific product was included.

### Step two: coding of on-package information

Images of all sides of each formula pack were obtained from supermarket or company websites or by visiting shops and photographing all sides of products. At least two sources were cross-referenced to ensure we collected accurate images of products currently available. Images were transferred to NVivo 12 for analysis.

**Fig. 1:**
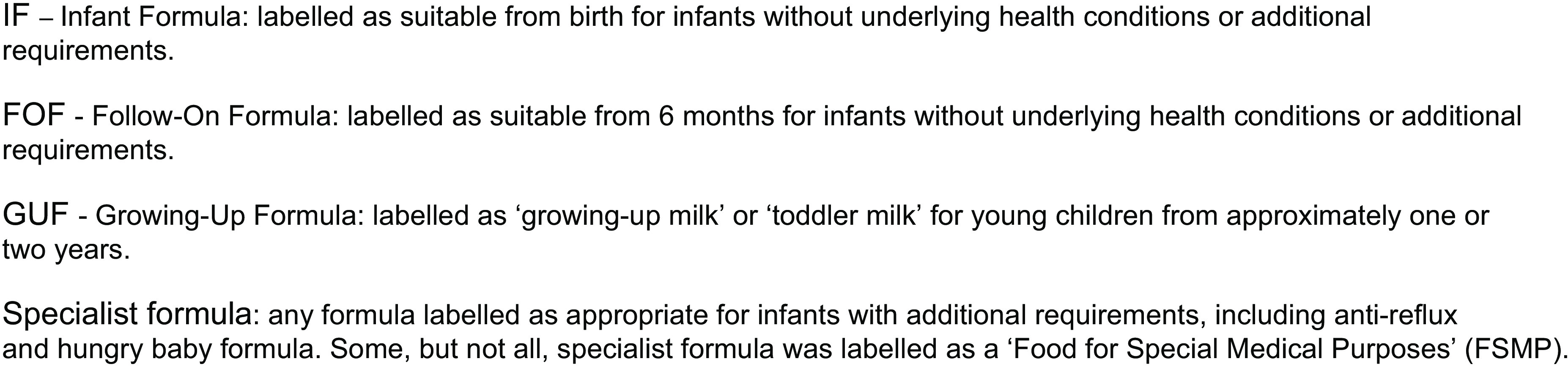
Classification of formula products

Text and images of formula products were explored using content analysis. Products were categorised as IF, FOF, GUF or specialist formula (Fig. 1). An initial coding framework was developed using a combined inductive and deductive approach (RC and SE). This framework drew on insights obtained from previous studies examining nutrition and health claims in formula adverts^(19,25)^, information from First Steps Nutrition Trust reports^(26,27,28)^, EU and GB regulatory documents^(5,6)^ and DHSC guidance^(14,29,30)^. New DHSC Guidance was not available at the time of analysis, but these are similar^(15)^. This approach facilitated the comparison of imagery and text on labels, with requirements set out in the legislation and DHSC guidance. The framework allowed for additional images and text to be explored and categorised into themes. RC and SE coded one product together to create an initial coding framework. They then coded three products each, independently, before reviewing each product and code together and modifying the framework. The coding framework, code book and specific examples were then discussed with the wider research team to achieve consensus. RC and SE then coded the remaining products independently and with regular discussions with the wider team to iteratively modify the framework. Legislation and guidance were continuously re-examined to ensure items relevant to these were identified. The framework was then refined, the codebook was edited and preliminary results, particularly regarding implied claims, were reviewed by the research team.

### Analysis of nutrition and health claims

Nutrition claims and health claims were defined according to existing legislation^(29,31,32)^. A nutrition claim was defined as any text or image thought to state, suggest or imply that a product had particular beneficial nutritional properties due to the nutrients or other substances it contained (e.g. ‘vitamin D’ in bold text on the front of pack). Nutrition claims appearing on IFs were counted as ‘nutrition claims considered non-permitted’ as legislation prohibits their use on IF. An exception was made for claims for DHA (an *n*-3 fatty acid), which are permitted on IF for a transition period if accompanied by a statement explaining that DHA addition to IF is mandatory. When a DHA claim was made on an IF, it was therefore considered permitted, and the pack was examined for the presence of the required statement about mandatory addition. Nutrition claims on FOF, GUF and formula labelled as FSMP were categorised as ‘nutrition claims considered non-permitted’ if the average consumer would be unlikely to understand them, as Regulation (EU) No 1924/2006 only permits the use of nutrition claims if the average consumer can be expected to understand the beneficial effects as expressed in the claim^(32)^.

Text or images, thought to state or imply a relationship existed between a product or one of its constituents and health, were coded as health claims. For example, ‘Calcium and vitamin D to support normal bone development’. Items coded as health claims were further categorised according to whether or not they appeared in the Great Britain Nutrition and Health Claims (GB NHC) Register. This database lists all ‘approved health claims’^(33)^. Any health claim identified on IF was categorised as a ‘health claims considered non-permitted’, as health claims are not permitted on IF.

Claims such as ‘suitable for milk intolerance’ were not counted as a nutrition or health claim on a FSMP as these products are required to state on the label which condition the product is suitable for and why it is suitable for the dietary management of this condition.

### Assessment of similarity between products

Legislation states IF and FOF should be easily distinguishable within a product range of the same brand. Similarities between IF and FOF packaging were evaluated to assess compliance with this stipulation using a derived ‘similarity score’. The similarity score was based on five features highlighted by DHSC^(14)^: colour of pack, size and position of logo, image on pack, position of image and position of product name. Products were assigned 0 if the feature differed between FOF compared to IF, 0·5 if similar and 1 if identical. A composite ‘similarity score’ between 0 and 5 was then produced for each FOF, with a higher score indicating a higher level of difficulty to distinguish FOF from the IF. The same procedure was followed to compare specialist formula with same-brand IF.

### Assessment of compliance with legislation referencing breastfeeding

All IF and FOF packs are legally required to display information about the superiority of breastfeeding under the heading ‘Important Notice’^(6)^. Compliance with this requirement was assessed by recording the presence of the Important Notice and describing its appearance, size and location on the pack. Legislation states that IF and FOF ‘shall not include pictures of infants, or other pictures or text which may idealise the use of such formula’^(5)^. DHSC Guidance was used to identify text and images that could be considered ‘non-permitted’^(14)^.

### Statistical analysis

A dataset was compiled in SPSS 27 to analyse formula product features quantitatively. A sub-sample of 20 % of products was double-coded by RC and SE independently, and interrater agreement was assessed using Cohen’s *κ*. Inter-rater reliability was high for the presence of both nutrition claims and health claims (*κ* = 100 %) and substantial for identifying the presence of a comparison to breast milk (*κ* = 70 %). As perfect agreement was not achieved, the code book was updated, and the two researchers then viewed all products independently to assess compliance with DHSC guidance regarding text which may suggest a similarity to breastmilk and use of images that may idealise the use of formula. Discrepancies were discussed with the wider team to reach consensus. Pearson’s Chi-square was used to explore differences between a total number of nutrition and health claims on IF, FOF and GUF (stages 3 and 4 combined).

## Results

Overall, seventy-one distinct formula products were identified, and 302 images analysed. Of these, fifty-five products were labelled as appropriate for individuals without underlying health conditions or additional requirements, comprising IF (*n* 18), FOF (*n* 18) and GUF (*n* 19). The remaining 16 were classified as specialist formulas. The main results are summarised in Table 1 alongside details from the relevant legislation and DHSC Guidance.

### Nutrition and health claims

Most on-package formula labels included both nutrition and health claims (Table 2). Analysis showed the total number of nutrition claims, and the total number of health claims, was smaller on IF compared with FOF and GUF (*P* < 0·01). A DHA claim was found on 94 % of IF. A statement about DHA being mandatory in IF was included on 100 % (*n* 14) of IF products required to include it. On thirteen of these fourteen IF products, the DHA claim appeared prominently on the front of pack and an asterisk was used to link this to the required statement about DHA addition being mandatory (e.g. ‘in accordance with legislation for all infant formula’). This mandatory statement was in smaller font in a different location, generally on the back of pack alongside other mandatory information. Three IF products contained hydrolysed protein, and at the time of product sampling, a statement about the mandatory nature of DHA addition had not yet come into force for these products. A small number of other nutrient claims were found on IF but these were likely due to older packaging still being used. All FOF and GUF included nutrition claims, with an average of 5·4 nutrition claims per pack. The most common nutrition claims were for *n*3s, vitamin D, Fe and Ca. Nutrition claims that could be considered non-permitted were found on 44 % of FOF and 52 % of GUF. These used highly technical terms considered unlikely to be understood by the average consumer, for example, 2’FL and LNnT.

**Table 2:** On-package nutrition and health claims on formula products (*n* 71)

	Infant formula (Stage 1)		Follow-on formula (Stage 2)		Growing-up formula (Stage 3)		Growing-up formula (Stage 4)		Specialist formula		All formula products	
	*n*	%	*n*	%	*n*	%	*n*	%	*n*	%	*n*	%
Number of products	18		18		16		3		16		71	
Products with a nutrition claim	17	94	18	100	16	100	3	100	11	69	61	93
Products with nutrition claim considered non-permitted (highly technical)	4	22	8	44	9	56	1	33	7	44	29	41
	Mean		Mean		Mean		Mean		Mean		Mean	
Number of nutrition claims/pack	1·6		5·4		5·5		5		1·3		3·5	
	*n*	%	*n*	%	*n*	%	*n*	%	*n*	%	*n*	%
Products with health claim in GB NHC Register	0	0	14	78	13	81	3	100	0	0	30	71
Products with health claim considered non-permitted as not in GB NHC Register	3	17	3	17	4	25	0	0	3	19	13	18
	Mean*		Mean*		Mean*		Mean*		Mean*		Mean*	
Number of claims considered as health claims/pack	0·2		2·5		2·6		3·0		0·2		1·4	

* Claims included in the GB NHC Register and those considered to be health claims but not on the GB NHC Register.

None of the IF packs included health claims from the GB NHC Register. However, three IF (17 %) included other statements considered to be health claims, for example ‘*contains all the nutrients your baby needs to grow if they are not being breastfed*’. IF products are not permitted to include health claims, thus these were coded as ‘non-permitted’ health claims. In addition to statements counted as non-permitted health claims, we found other statements that were not clear health claims but may be understood in a similar way. For example ‘*We have been leading research in baby nutrition for over 100 years and have produced brand X First Infant Milk, a nutritionally complete breast milk substitute, expertly created with nature in mind to support babies’ unique nutritional needs*’. As it was not possible to define what constituted an implied health claim, their frequency could not be recorded. However, messaging relating to concepts such as completeness, meeting babies’ needs, development and progress were widespread.

The majority of FOF (78 %) included permitted health claims from the GB NHC Register. Health claims for bone, cognitive and visual development were found in 72 % of FOF (*n* 14/18). Health claims relating to physiological processes (e.g. functioning of the immune system) were included on 61 % of FOF (*n* 11/18). GUF were found to include a similar number and type of health claims to FOF. In addition, some GUF included claims that would not be permitted on IF or FOF as they claimed a similarity to breastmilk, for example, ‘*brand X Growing Up Milk now contains 2’FL which is structurally identical to the most abundant oligosaccharide found in breast milk*’. GUF products were found to be almost identical in appearance to the brand’s FOF, which also claimed to include 2’FL. However, the FOF did not include any explanation of what 2’FL was.

### Specialist formulas

We identified specialist formulas (*n*16) which included seven products labelled as a FSMP: three ‘Comfort’ formulas for infants with colic and four anti-reflux formulas. Only two of seven FSMP displayed the required phrase ‘to be used under medical supervision’, and this was in small lettering on the back of the pack. The other five FSMP suggested asking for medical advice before use, again in small lettering. The nine specialist products not labelled as FSMP included one ‘Comfort’ formula, four ‘Hungry’ formulas, two lactose-free formulas, one soya based and one hydrolysed protein formula claiming to reduce the risk of developing an allergy to cows’ milk protein. None of these nine formulas included information regarding the need for medical supervision.

### Similarity between infant formula, follow-on formula and specialist formulas

Of the eighteen FOF packs identified, 72 % had a ‘similarity score’ ≥4 out of 5 (mean 4·4 (sd 0·65)), indicating a high degree of similarity with IF products. Overall appearance, including colours, text and images on many IF and FOF products were near identical. Other products had notable similarities, for example, a baby polar bear lying down on the IF but sitting on the FOF, suggesting a progression. Likewise, specialist formulas were presented in packaging similar to IF, with two FSMP (28·6 %) and five other specialist products (55·6 %) having a ‘similarity score’ ≥4 out of 5 (mean score 3·6 (sd 1·02) and 4·2 (sd 0·71), respectively).

### Messaging relating to breastfeeding

All formula packs (*n* 71) included a statement concerning the superiority of breastfeeding, as required by legislation. Despite DHSC guidance that this statement ‘*should be afforded a high degree of prominence*’^(14)^ it was generally in the smallest lettering on the pack and hidden at the back. In contrast, the use of text and images that might idealise the use of BMS or suggest a product was equivalent to breast milk were widespread. One IF (6 %) included text that DHSC guidelines^(14)^ suggest may be understood to imply a product is closer to or inspired by breastmilk, ‘*Our expert team at [Brand X] nutrition is dedicated to understanding the complex structure of breast milk and applying the learnings from nature to our own products’*. Similar phrases, which may breach legislation, were found on 22 % of FOF, 10 % of GUF and 6 % of specialist formulas. Images which may idealise the use formula, and therefore be considered non-permitted, were found on 67 % of IF, 78 % of FOF, 68 % of GUF and 75 % of specialist formulas. These images included teddy bears, baby elephants, a stork carrying a baby rabbit and a stylised image of a mother and infant.

### Messaging not covered by current legislation

Promoting follow-on products within a product line was common. The labels of 94 % of IF packs showed an advertisement for FOF and 39 % included an advertisement for GUF. A quarter (27 %) of IF packs included nutrition and health claims within an advertisement for FOF or GUF. Similarly, 78 % of FOF included advertisements for GUF. Most of the nineteen GUF were labelled as suitable from ‘12 months’ of age. However, five GUF were labelled as ‘organic’ and suitable from the ‘12th month’.

When exploring further features of packs, not covered by the legislation, themes relating to science, nature and emotional support were identified. All BMS packs (100 %) cited scientific and expert involvement in their formulation. Many also used scientific imagery, such as pictures resembling molecules. Text and images implying natural products were also common, such as ‘*Feeding life with pure nature*’. This text was often accompanied by images of cows, goats or rural scenery. Many products used caring and emotional language. In total, 72 % of packs referred to love, care or support for parents or babies, for example, ‘*we believe love and care can help when looking after your little one and we’re here for you on your journey*’. This was often followed by signposting to the brand’s other promotional channels. The majority (68 %) of packs provided information about the brand’s website or telephone line, including parent clubs and support lines run by ‘*experts and experienced mums*’.

## Discussion

Analysis of on-package formula marketing of seventy-one formula products in GB identified widespread use of health and nutrition claims, and other text and images used for promotion. DHSC guidance on the GB legislation was often not followed when labelling formula products for sale in GB. Nutrition and health claims that could be considered non-permitted were found on all types of formula – IF, FOF, GUF and specialist formula. As GB BMS legislation does not provide the same level of detail as found in the DHSC guidance, the research team found it challenging to identify whether or not the labelling on BMS products breached the law^(14)^.

We found that detailed DHSC guidance was not followed, and both text and images that could be considered to promote BMS are widely used; this finding supports and highlights the importance of the WHO call for loopholes in the legislation to be closed to strengthen enforcement and implementation of legislation restricting BMS promotion^(35)^. Consumers have been shown to be confused by nutrition and health claims on formula and certain claims appear to provide a ‘health halo’ effect, increasing the perception that a product is healthy and discouraging consumers from looking on other sides of the pack^(36)^. At the same time, these claims may also directly increase the intention to purchase^(37)^. Nutrition and health claims are recognised as promotional tools in the legislation. A jump in claims was seen from IF to FOF, with FOF including an average of 5·4 nutrition claims and 2·5 health claims per pack. This jump and the clear similarity between IF and FOF packaging provide additional evidence to support calls for marketing restrictions to apply to FOF as well as IF to prevent them being used by BMS companies to circumvent restrictions on IF promotion^(4)^. Claims for nutrients, such as Fe, that must be included in all FOF by law do not inform consumers’ choices. Claims for nutrients that are not added to all FOF are likewise unhelpful to consumers as ingredients would be added to all formulas if good evidence of benefit existed^(39)^. These additions are also unlikely to be understood. We found 44 % of FOF and half of GUF included claims for little-known nutrients, such as 2’FL (an oligosaccharide). Oligosaccharides are considered a non-essential addition to IF and FOF by the European Food Safety Agency,^(38)^ and there is concern that the use of such unsubstantiated claims may undermine breastfeeding^(39)^. By indicating a product contains 2’FL and also that 2’FL is found in breastmilk, it leads the consumer to infer the product compares favourably with breastmilk, an advertising strategy called ‘probabilogic’ which Berry describes formula companies use to position their brand with regard to the most important competitor – breastmilk^(40)^. While claims that are unlikely to be understood are not permitted, the legislation does not list examples of such claim or nutrients that are likely to be (or not) understood. More clarity in the legislation surrounding this matter would avoid confusion.

The finding that both the mandatory statement regarding DHA addition and the mandatory ‘Important Notice’ highlighting the superiority of breastfeeding were small and difficult to locate, is not surprising. While the legislation states that this information must be included, it does not provide specifics regarding placement or appearance. DHSC guidance advises that the important notice should be clearly visible and prominently displayed but also lacks specific detail. Mandatory information is often relegated to the back of pack and crowded together, meaning it is unlikely to be noticed^(41)^. Legislation specifying the appearance of mandatory information on formula, including the minimum percentage of pack that should contain a given piece of information, is one way of ensuring greater prominence. Another aspect of the legislation where lack of detail was problematic was in the identification of text and images idealising the use of formula. Both text and images that DHSC guidance suggest may be considered non-permitted, because of idealising formula, was common, but the legislation itself doesn’t list what is and isn’t permitted, making it difficult to enforce. This adds to growing concern that existing legislation is inadequate as it leaves room for ‘creative compliance’^(19)^ and needs a drastic overhaul to protect child health and maternal autonomy rather than industry expansion^(35,41)^.

We found widespread use of implied claims suggesting particular products were superior as they were closer to breastmilk than others. For example, ‘*Our breastmilk research has enabled us to develop our next generation brand X follow on milk*’. This is a concern as claims of this nature have been shown to influence both beliefs and purchasing behaviours^(16)^. Marketing messages such as these were widespread and have been described by the WHO and the United Nations children’s agency (Unicef) as ‘slick and misleading’^(35)^.

Our analysis showed same brand IF and FOF packs were difficult to distinguish, despite legislation stating they should be designed to enable clear distinction^(5)^. Similarity across product ranges is used for cross-promotion^(39)^ and is well recognised. Indeed, the NHS warns caregivers ‘*The labels on follow-on formula look very similar to those on first infant formula. Read the label carefully to avoid making a mistake*.’^(10)^. Research in Australia^(42)^ and Italy^(43)^ has shown pregnant women, and new mums may only see advertisements for formula for older infants due to restrictions over advisements for IF, but they often struggle to make a distinction and report having seen IF advertised due to the use of consistent design features which are used to build a strong brand identity^(42)^. The similarity between same brand IF and FOF also extends to GUF, although these were not assessed systematically in this research. As well as facilitating cross-promotion, this helps convey the message that infants should progress from one product to the next, which is not consistent with healthy eating guidelines in GB to breastfeed or use either IF (up to 1 year of age) or cow’s milk (from 1 year of age). The perceived need to use formula beyond a year is reinforced by advertisements for FOF and GUF, on the majority of IF packs. The inclusion of nutrition and health claims and claims regarding a similarity to breastmilk found within GUF advertisements shown on packs of IF is another covert form of advertising, which is likely to have a halo effect and promote IF use.

GUF are the fastest-growing sector of the formula industry with brands aiming to keep customers buying their products beyond infancy^(34)^. GUF packaging is similar to IF and FOF but is not regulated in the same way. This means that GUF packaging also acts as a means of promoting IF and FOF. There is concern over the increasing use of GUF, the lack of regulation and the aggressive marketing techniques used to promote them^(44)^. GUF contain higher levels of free sugar than cow or breast milk, which are the recommended milks from 12 months, and there is no evidence that they provide extra nutritional benefits for young children^(18)^. An unexpected finding regarding GUF was that while most were labelled as suitable from ‘12 months’, some stated ‘12th month’ so that they could still be labelled as organic. These products would not be permitted to be labelled as organic if they were labelled as suitable from ‘12 months’ because food products are prohibited from organic classification if they are fortified with nutrients that are not required by legislation, as is the case for GUF. The adjustment in product age recommendation, while suiting brands who want to market their products as organic, may confuse consumers and is another example of brands being allowed to self-govern.

While the legislation makes a clear distinction between formula labelled as a FSMP and other formula (IF and FOF), our analysis revealed no clear difference. We found similar images and packaging on FSMP products despite the need for such products to be used only under medical supervision. Three ‘Comfort’ formulas, marketed for infants with colic, were labelled as a FSMP and another ‘Comfort’ formula was labelled as IF. Among the specialist formula not labelled as a FSMP, we found one product labelled as suitable for cow’s milk protein allergy and another for lactose intolerance. These two conditions require completely different dietary management and could be misdiagnosed and inappropriately managed without medical supervision. The wisdom of allowing brands to choose whether a formula is a FSMP and needs to be used under medical supervision or not is questionable. Whether or not specialist products should be available over the counter at all has been challenged^(45)^ and there is concern that claims for FSMP formulas are not justified by the evidence^(46)^ and that products are currently overused and misused^(47)^.

The widespread use of emotional messaging has previously been highlighted as a major strategy used by the multinational BMS industry, whereby they pitch themselves as a source of support and friendship^(34)^. The frequent use of imagery relating to the natural environment to sell BMS has also been reported in a Chinese study of parenting apps^(25)^. Findings that some products concentrate on scientific imagery and claims, while others include pictures of teddy bears and highlight parenting clubs and support, are consistent with the idea of bespoke marketing to target caregivers, particularly mothers, with different profiles^(34)^. Our findings relating to ubiquitous references to science, nature and use of emotional and supportive statements are in line with findings reported by the WHO and UNICEF report, in which they describe these as manipulative marketing tactics that take advantage of parents’ anxieties and aspirations^(35)^. Many of the claims relating to research and products being the most advanced scientifically, were in line with those described by Hastings *et al.* as essentially meaningless, but effective in reassuring parents feeling guilty about not breastfeeding^(34)^.

Our study has some limitations. A small number of the products included in this analysis included older versions of packaging which had not yet been updated to comply with the most recent legislation. When more than one version of a product was available, the most up to date version was analysed. Some products were coded by two researchers, but an element of subjectivity remained in the identification and classification of marketing messages. However, our interpretation appeared fair as the potentially non-permitted health claims we identified were the same as those appearing in updated DHSC guidance^(15)^, which were released after this analysis was completed – for example, ‘*contains all the nutrients your baby needs to grow strong and healthy*’, ‘*easy to diges*t’ and ‘*gentle*’. This analysis however is the first to systematically assess a wide range of on-package labelling elements, including text and images, providing a comprehensive overview of how formula products are labelled in GB.

In conclusion, guidance provided by DHSC was not observed, and compliance with BMS legislation was difficult to assess. Results suggest that additional detail is needed in BMS legislation regarding text and images that can and cannot be used. If certain text or information is mandatory, then positioning and size should be specified in the legislation. This is to avoid information being relegated a position where it is unlikely to be seen. Legislation should also be extended to include GUF products and to ban the use of certain marketing practices, such as the inclusion of FOF advertisements and claims on IF. More effective legislation regarding on-package labelling of formula is an essential component of policy to protect the public from inappropriate marketing and undue commercial influence.

## Supplementary material

For supplementary material accompanying this paper visit https://doi.org/10.1017/S1368980023000216. click here to view supplementary material
