# Supplementary material for: Content analysis of on-package formula labelling in Great Britain: use of marketing messages on infant, follow-on, growing-up and specialist formula
Source: Public Health Nutr. 2023 Jan 30;26(8):1696–705. doi: 10.1017/S1368980023000216 (PMC10427444; doi:10.1017/S1368980023000216)
Supplement: Supplementary file 1 [file S1368980023000216sup001.docx]

**Supplementary material – Identification of formula products for analysis**

Formula products available over the counter were identified by:

1. Searching retailers websites using the following terms: “follow-on formula”, “toddler milk”, “growing-up milk” “growing-up”, “baby milk”, “formula”, “infant formula” and “baby food”.

Retailers websites searched:

- The 10 supermarkets with the largest market share^1^ (Tesco, Sainsbury’s, Asda, Morrisons, Aldi, Co-op, Lidl, Waitrose, Iceland and Ocado).
- The four pharmacy chains with the largest market share^2^ (Boots, Lloyds, Bestway, Superdrug).

2. Checking the websites for formula brands identified (Aptamil, Cow & Gate, SMA nutrition, Hipp Organic, Kendamil, Nannycare).

3. Examining Kantar household purchasing data for September 2016 to March 2020 were also examined (Institute of Fiscal Studies calculations based on Kantar FMCG Purchase Panel).

4. Reading information from First Steps Nutrition Trust^3^.

1. Kantar World Panel. Grocery Market Share - Kantar. Accessed August 17, 2020. https://www.kantarworldpanel.com/grocery-market-share/great-britain/snapshot/12.07.20/

2. Dispensing Chemists in the UK - Industry Data, Trends, Stats | IBISWorld. Accessed August 18, 2020. https://www.ibisworld.com/united-kingdom/market-research-reports/dispensing-chemists-industry/

3. First Steps Nutrition Trust. Background information about infant milks The infant milk market in the UK. Accessed May 12, 2020. https://static1.squarespace.com/static/59f75004f09ca48694070f3b/t/5e84513ccb44b3644a86ff26/1585729852406/The_infant_milk_market_in_the_UK.pdf
